# Cost-effectiveness analysis of lumacaftor and ivacaftor combination for the treatment of patients with cystic fibrosis in the United States

**DOI:** 10.1186/s13023-018-0914-3

**Published:** 2018-09-29

**Authors:** Dolly Sharma, Shan Xing, Yu-Ting Hung, Rachel N. Caskey, Maria L. Dowell, Daniel R. Touchette

**Affiliations:** 1 Department of Pharmacy Systems, Outcomes & Policy, College of Pharmacy, University of Illinois at Chicago, Chicago, IL USA; 2 Departments of Internal Medicine and Pediatrics, University of Illinois at Chicago, Chicago, IL USA; 3 Section of Pulmonary and Sleep Medicine, Department of Pediatrics, The University of Chicago, Chicago, IL USA

**Keywords:** Cost-effectiveness, Cystic fibrosis, Lumacaftor, Ivacaftor

## Abstract

**Background:**

Lumacaftor/ivacaftor was approved by the Food and Drug Administration (FDA) as a combination treatment for Cystic Fibrosis (CF) patients who are homozygous for the F508del mutation. The objective of this study was to assess the cost-effectiveness of lumacaftor/ivacaftor combination for the treatment of CF homozygous for F508del CF Transmembrane Conductance Regulator (CFTR) mutation.

**Methods:**

A Markov-state transition model following a cohort of 12 year-old CF patients homozygous for F508del CFTR mutation in the United States (US) over two, four, six, eight and ten years from a payer’s perspective was developed using TreeAge Pro 2016. Markov states included: mild (percentage of predicted forced expiratory volume in 1 s or FEV1 > 70%), moderate (FEV1 40–70%), severe (FEV1 < 40%) disease, post-transplant, and death. Pulmonary exacerbation and lung transplant were included as transition states. All the input parameters were estimated from the literature. A 1-year cycle length and 3% discount rate were applied. To assess uncertainty in long-term treatment effects, several scenarios were modelled: 100% long-term effectiveness (base-case), defined as improvement in FEV1 in the first year followed by no annual FEV1 decline and a constant reduction in pulmonary exacerbations throughout, 75%, 50%, 25% and 0% (worst case) long-term effectiveness, where treatment effects were intermediate from the second year of treatment until the end of the time horizon. Other scenarios included changing the starting age of the cohort to 6 and 25 years. Primary outcome included incremental cost-effectiveness ratio (ICER) in terms of cost per quality adjusted life year (QALY) gained. One-way and probabilistic sensitivity analyses were performed to determine uncertainty.

**Results:**

Under the base-case, Lumacaftor/ivacaftor resulted in higher QALYs (7.29 vs 6.84) but at a very high cost ($1,778,920.88) compared to usual care ($116,155.76) over a 10-year period. The ICER for base-case and worst-case scenarios were $3,655,352 / QALY, and $8,480,265/QALY gained, respectively. In the base-case, lumacaftor/ivacaftor was cost-effective at a threshold of $150,000/QALY-gained when annual drug costs were lower than $4153. The results were not substantially affected by the sensitivity analyses.

**Conclusions:**

The intervention produces large QALY gains but at an extremely high cost, resulting in an ICER that would not typically be covered by any insurer. Lumacaftor/ivacaftor’s status as an orphan drug complicates coverage decisions.

## Background

CF is a rare lethal genetic disease that affects over 30,000 people in the US with about 1000 newly diagnosed individuals each year. The condition is caused by mutations in the CF Transmembrane Conductance Regulator (CFTR) gene leading to deficiency and/or defects in the CFTR proteins [1]. Out of the 1800 mutations found in CFTR gene, the most common is the F508del, which impacts 86.5% of patients with CF in the US CF Foundation Patient Registry [2]. About half of patients with CF are homozygous for this allele. Despite the advances in drug therapies in the US, the median predicted survival varies from a low of 37 years (based on 2010 mortality levels), to an upper bound of 50 years (based on extrapolation of improvements in survival between 2000 and 2010) [3]. Obstructive lung disease is the major cause of morbidity and mortality in CF accounting for 80% of deaths. As the disease progresses, patients require more intensive treatment and healthcare services, including more frequent and prolonged hospital admissions and lung transplant [2].

The U.S. Food and Drug Administration (FDA) approved lumacaftor/ivacaftor as a combination treatment on July 2, 2015 for patients 12 years and older who are homozygous for the F508del mutation. This is the first therapy that targets and partially corrects the primary defect in this mutation [4]. The two phase 3 studies, TRAFFIC and TRANSPORT, reported statistically significant improvements in the absolute change in percentage of predicted forced expiratory volume in 1 s (FEV1) by 2.6 to 4% and a reduced rate of pulmonary exacerbations by 30 to 39% with lumacaftor/ivacaftor treatment compared to placebo at the end of 24 weeks [5].

Given that CF affects fewer than 200,000 individuals in the US, lumacaftor/ivacaftor was approved receiving orphan drug status. The number of treatments available for rare diseases have increased substantially after the enactment of the Orphan Drug Act (ODA) of 1983, which lowered the barriers for development of drugs for rare diseases and provided incentives that increased revenues post-approval by decreasing competition. However, prices of these drugs can amount to annual costs of hundred to thousand dollars, which poses a substantial burden on insurers [6]. Traditionally, payers have covered orphan drugs owing to the small patient populations using these drugs; however, with accelerating new launches and growing pipeline of such drugs, the payer concerns and scrutiny regarding costs of these drugs are increasing [7]. This economic pressure might shift to patients resulting in higher cost sharing and decreased access to these drugs by patients, often who cannot afford the increased out of pocket expenses [8].

Given only short-term evidence for lumacaftor/ivacaftor effectiveness and its high cost (estimated to be about $259,000 per year), it is important to evaluate the cost-effectiveness of this drug to better inform payer and policy decision making [9]. A previous study examined the lifetime health and economic outcomes associated with lumacaftor/ivacaftor use in a 25 year-old CF population in the US [10]. However, lumacaftor/ivacaftor was initially approved for use in patients aged 12 years and older. This approval was subsequently updated in 2016 to patients aged 6 years and older. Other important limitations of this prior analysis were that the impact of lumacaftor/ivacaftor on pulmonary exacerbations was not considered, transition probabilities were based on Australian data, and a 90% reduction in cost was assumed after patent expiration. Given the differences in disease burden in younger patients with this progressive condition, potential differences in a U.S. population, and unlikely occurrence of significant reductions in the cost of lumacaftor/ivacaftor, there was a need to reevaluate the potential long-term benefits and cost-effectiveness of lumacaftor/ivacaftor [11].

Thus, the primary objective of this study was to assess the cost-effectiveness of lumacaftor and ivacaftor combination for the treatment of 12 year-old patients with CF homozygous for F508del CFTR gene compared to usual care in the US from a payer’s perspective at a willingness to pay (WTP) threshold of $150,000 per quality adjusted life year (QALY) gained over a time horizon of two, four, six, eight and ten years. We also aimed to assess the cost-effectiveness of the intervention in 6 and 25 year old and under different efficacy scenarios to model the uncertainty associated with long-term treatment.

## Methods

A Markov state transition model was developed using TreeAge Pro 2016 to evaluate the outcomes associated with or without treatment with lumacaftor/ivacaftor in 12 year-old patients with CF in the US from a payer’s respective and following the recommendations of the Second Panel on Cost-Effectiveness in Medicine [12]. Health outcomes and costs included in our analysis are presented using the panel’s Impact Inventory Template in Table 1.

**Table 1:** Checklist of health outcomes and costs included under payer’s perspective as per the Impact Inventory Template given by Second Panel on Cost-Effectiveness in Health and Medicine

Type of Impact (list category within each sector with unit of measure if relevant)	Included in This Base Case Analysis From payer’s perspective (Yes/No)
Health outcomes (effects)	
Longevity effects	Yes
Health-related quality-of-life effects	Yes
Other health effects (eg, adverse events and secondary transmissions of infections)	No
Medical costs	
Paid for by third-party payers	Yes
Paid for by patients out-of-pocket	No
Future related medical costs (payers)	Yes
Future related medical costs (patients)	No
Future unrelated medical costs (payers)	No
Future unrelated medical costs (patients)	No

### Model structure and patient population

**Fig. 1:**
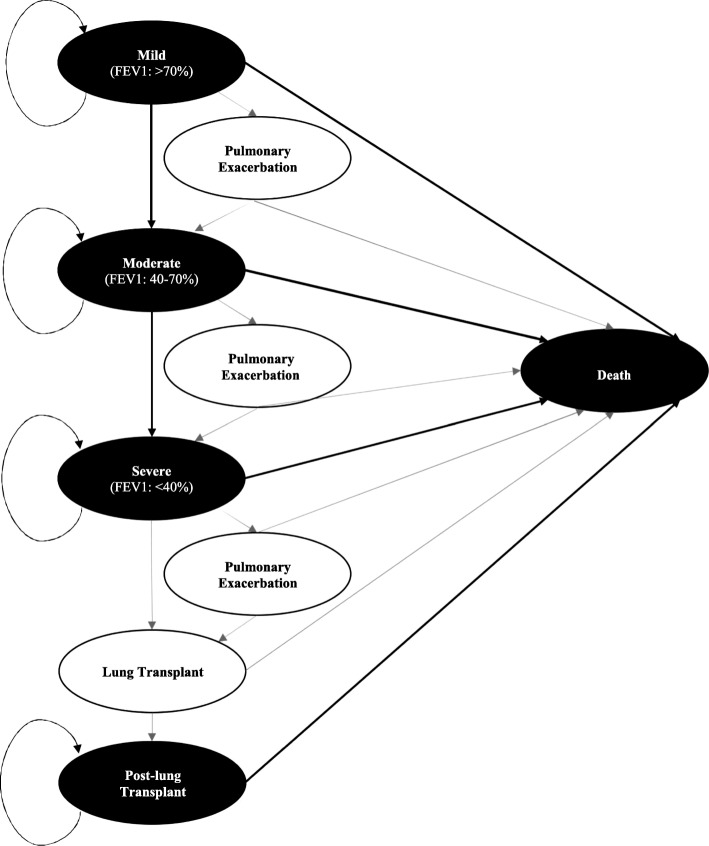
Markov model included five health states: mild (FEV1 > 70%), moderate (FEV1 40–70%), severe (FEV1 < 40%), post-transplant and death and two transition states: pulmonary exacerbation and lung transplant. Transitions to improved states were not allowed for the usual care arm. In some scenarios, transitions to improved states were allowed for the treatment arm

Treatment-naïve 12 year-old patients with CF were modeled to receive either usual care or usual care and lumacaftor/ivacaftor. Usual care comprised of treatment with antibiotics, pancreatic enzymes, aminoglycosides (inhaled tobramycin as well as intravenously administered aminoglycosides) and DNase [13]. The model was based on the natural progression of CF and included five health states (Fig. 1): 1) mild disease (FEV1 > 70%), 2) moderate disease (FEV1 40–70%), 3) severe disease (FEV1 < 40%), 4) post-lung transplant and, 5) death. The distribution of patients within various stages of disease was based on natural prevalence of the disease at age 12 as reported in the CF Foundation Patient Registry annual report (87% in mild, 11% in moderate, 2% in severe) [2]. In addition, two transition states were allowed to occur within a cycle: 1) pulmonary exacerbations requiring intravenous antibiotics and/or inpatient stay, and 2) lung transplant. Although data showed that multiple pulmonary exacerbations were possible within each model cycle, multiple exacerbations were not allowed in a single model cycle. Instead, costs were weighted to account for increased costs associated with more than one exacerbation within a model cycle. In addition, a mortality rate of 0.1% associated with pulmonary exacerbation in mild and moderate state was assumed based on expert opinion of one of the authors (MLD), an associate director of the University of Chicago’s Cystic Fibrosis Center. Patients with severe disease could eventually require a lung transplantation moving to a post-transplant state. Although possible, multiple lung transplants were not allowed in the model as it is a rare scenario. The model incorporated CF population specific mortality. A constant cycle length of 1 year was used and the model was run for a time horizon of 10 years. A time horizon of 10 years was chosen considering the lack of long-term effectiveness data and the continued development of newer therapies for CF. Considering the impact of newer therapy options, it is likely that therapy will change dramatically over the next 10 years.

### Clinical input parameters

Systematic reviews of the literature were conducted in PubMed to identify clinical trials, observational studies, and economic evaluations that could be used for estimation of input variables, by using the specific search terms “cystic fibrosis,” and “pulmonary exacerbation” and “lung transplant” and their combinations, limited to English language and publications between 2005 and June, 2018. The references of published cost-effectiveness analyses and relevant reviews were also reviewed. Eligible full text studies were reviewed by three independent reviewers and relevant data was extracted for populating model probabilities associated with clinical outcomes associated with CF and related treatments. All the probabilities used in the model are shown in Table 2.

**Table 2:** Summary of key model inputs: probabilities, costs and utilities. Transition probabilities are presented for each model transition, including those from a Markov state (e.g. Mild CF) to transition states (e.g. Pulmonary Exacerbation)

Variable	Base-case	One-way		Source
		Low	High	
Probabilities				
Mild to Death	Age-specific mortality for CF population			[3]
Mild to Pulmonary Exacerbation	0.3213	0.2571	0.3856	[16]
Pulmonary Exacerbation in Mild to Death	0.001	0.0008	0.0012	Assumption
Pulmonary Exacerbation in Mild to Moderate	0.0697	0.0558	0.0837	[14, 15]
No Pulmonary Exacerbation in Mild to Moderate	0.0307	0.0245	0.0368	[14, 15]
Moderate to Death	Age-specific mortality for CF population			[3]
Moderate to Pulmonary Exacerbation	0.5748	0.4599	0.6898	[16]
Pulmonary Exacerbation in moderate to Death	0.001	0.0008	0.0012	Assumption
Pulmonary Exacerbation in Moderate to Severe	0.0617	0.0493	0.074	[14, 15]
No Pulmonary Exacerbation in Moderate to Severe	0.0279	0.0223	0.0335	[14, 15]
Severe to Death	Age-specific mortality for CF population			[3]
Severe to Pulmonary Exacerbation	0.6794	0.5435	0.8153	[16]
Pulmonary Exacerbation in severe to death	0.2174	0.1739	0.2609	[18]
Transplant to Death	0.297	0.2376	0.3564	[17]
Pulmonary Exacerbation in Severe to Transplant	0.078	0.0624	0.0937	[17]
No Pulmonary Exacerbation in Severe to Transplant	0.078	0.0624	0.0937	[17]
Post-transplant to Death	Calculated year-wise			[19]
Lumacaftor/Ivacaftor:				
Moderate to Mild without Pulmonary Exacerbation	0.0935	0.0748	0.1123	[5, 14, 15]
Severe to Moderate without Pulmonary Exacerbation	0.5517	0.4414	0.6621	[5, 14, 15]
Moderate to Mild with Pulmonary Exacerbation	0.1045	0.0836	0.1254	[5, 14, 15]
Severe to Moderate with Pulmonary Exacerbation	0.5455	0.4364	0.6545	[5, 14, 15]
Relative risk for Pulmonary Exacerbation (Lumacaftor/Ivacaftor vs Placebo)	0.74	0.592	0.89	[5]
Costs (USD-2016)				
Lumacaftor/ivacaftor	188,660.43	150,928.34	226,392.51	[9, 20]
Mean cost of Pulmonary Exacerbation in Mild	2575.97	2575.97	30,949.79	[13]
Mean cost of Pulmonary Exacerbation in Moderate	8371.90	8371.90	41,969.47	[13]
Mean cost of Pulmonary Exacerbation in Severe	52,646.40	52,646.40	123,385.36	[13]
Mild	7566.91	5121.17	30,267.65	[13]
Moderate	9981.89	6709.60	39,927.54	[13]
Severe	17,226.80	8688.16	68,907.21	[13]
Transplant	1,056,002.23	844,801.78	1,267,202.67	[21]
Post-transplant	87,945.85 (1st year)	70,356.68	105,535.02	[21, 22]
	86,332.56 (2nd year onwards)	69,066.05	103,599.07	[22]
Utilities				
Mild	0.86	0.77	0.94	[23]
Moderate	0.81	0.72	0.89	[23]
Severe	0.64	0.57	0.7	[23]
Post-transplant	0.83	0.74	0.91	[23]
Pulmonary Exacerbation	− 0.17	− 0.15	−0.19	[23]

#### Efficacy of lumacaftor/ivacaftor combination (TRANSPORT and TRAFFIC)

The efficacy data of lumacaftor/ivacaftor was derived from two phase 3, randomized, double-blind, placebo-controlled studies: TRAFFIC and TRANSPORT, conducted to assess the effects of lumacaftor/ivacaftor in 12 year-old patients with CF, homozygous for the F508del CFTR mutation. Patients were randomly assigned to receive either lumacaftor (600 mg once daily or 400 mg every 12 h)/ ivacaftor (250 mg every 12 h) combination or matched placebo for a duration of 24 weeks to determine the absolute change in the percentage of predicted FEV1 from baseline at week 24 and change in rate of pulmonary exacerbations. At week 24, the results showed significant improvements in the mean absolute FEV1% ranging from 2.6 to 4.0%. Furthermore, the pooled analysis reported a reduction of 34–39% in the rate of pulmonary exacerbations in the treatment arm compared to placebo at week 24 [5].

To model the long-term efficacy of lumacaftor/ivacaftor, two main scenarios were analyzed. The base-case was constructed to consider a best case scenario (100% efficacy), in which it was assumed that initial lumacaftor/ivacaftor benefits observed in the clinical trial would be maintained while on therapy i.e. patients would not progress to more severe states while on lumacaftor/ivacaftor and a constant risk ratio for reduction in pulmonary exacerbation was applied throughout the duration of therapy. In the second scenario or the worst-case (0% efficacy), improvement in FEV1 levels was allowed only for the first year. For the second through 10th year, a constant decline similar to the usual care arm was assumed and no reduction in pulmonary exacerbations was considered. For the usual care and second year onward for the lumacaftor/ivacaftor strategies, improvement in FEV1 resulting in transitions to a better state were not allowed (e.g. from moderate disease to mild disease). The 48 week-exacerbation rate per patient and 24 week-proportion of exacerbation free patients for the treatment and placebo arms were used to obtain 1 year probabilities which were then used to derive the 1-year relative risk (treatment vs usual care) of having any pulmonary exacerbation compared to no exacerbation.

#### Disease severity health states and pulmonary exacerbations

Due to the lack of availability of data on mean FEV1 levels for 12-year-old patients with CF, mean FEV1 levels of 15 year-old patients with CF derived from literature were used to construct a random sample of 1000 patients with CF [14]. By using a bootstrapping method, annual FEV1 decline rates (with or without exacerbation) derived from the literature were applied to this sample to predict the progression of this population from 12 to 22 years of age (Table 2) [15]. Based on the distribution of the population in mild, moderate and severe state of disease for each age group, transition probabilities for disease progression from mild to moderate and moderate to severe health states were calculated. The probabilities for pulmonary exacerbation in mild, moderate and severe health states for the usual care arm were obtained directly from literature [16].

#### Lung transplantation and post-lung transplant

Age-specific probabilities of lung transplantation in the CF population were calculated from the data in the US CF Foundation Patient Registry annual report 2016 [17]. Patients in the treatment arm were not allowed to receive lumacaftor/ivacaftor post lung-transplant.

#### Mortality

Age-specific mortality in CF population was obtained from a study conducted by MacKenzie et al. [3]. Risk of mortality associated with pulmonary exacerbation in severe health state was obtained from a study conducted by Ellafi et al. [18]. Mortality associated with lung transplant was calculated from data in US CF Foundation Patient Registry annual report 2016 [17]. Age-wise probability of death was calculated as the cohort progressed in post-transplant states using the estimates from Stephenson et al. [19].

### Costs and utilities

Costs and utilities entered in the model were estimated by conducting a systematic search of published literature on CF related costs and quality of life. The cost of lumacaftor/ivacaftor was calculated as 72% of the average wholesale price (2015) listed by the manufacturer, as the net payments to insurers in the United States in 2014 have been reported to average 72% of gross payments [9, 20]. Payer specific direct costs were used to follow a payer’s perspective (Table 1) [11]. A study by Lieu et al. was used to determine costs associated with mild, moderate and severe disease states and pulmonary exacerbations [13]. As this cost study does not explicitly state costs as being related to exacerbations or non-exacerbation costs of care, we made an assumption that the costs of treating pulmonary exacerbations were comprised of hospitalizations and outpatient antibiotic treatments. All remaining costs (clinic costs, DNase, and pancreatic enzymes and other medications) were assumed to represent the annual costs of maintenance for having mild, moderate, or severe disease. Cost estimates for lung transplant included double-lung transplant.. These costs were derived from Millennium report and year-wise post-lung transplant costs were calculated using ratios from a study conducted by Dayton 2005 [21, 22]. The utilities and disutility associated with various health states and transition states were derived from a single modeling study conducted by Tappenden et al. [23]. Costs and utilities used in the model are shown in Table 2. All costs were inflation adjusted to 2016 prices when appropriate using the Personal Consumption Expenditure health component price index and a discount rate of 3% was applied to all clinical and economic outcomes for all the analyses [24].

### Outcomes and analyses

The primary outcome was ICER calculated as incremental cost per QALY gained associated with lumacaftor/ivacaftor treatment compared to usual care. The secondary outcomes were the total costs and QALYs generated with each treatment and the proportion of patients remaining in each health state over a period 10 years. The base-case analysis was also run for a period of two, four, six, and eight years to account for discontinuation of lumacaftor/ivacaftor and switching to newer drugs into the market. Additionally, several other scenario analyses were conducted, detailed below.

#### High-cost scenario

As the care of CF has advanced substantially since the Lieu et al. study and may have outstripped inflation over that period, results were tested in a high-cost scenario analysis by inflating the mild, moderate and severe state costs by four times of the cost included in the base case, resulting in costs approximating those presented in a more recent abstract [25]. Higher pulmonary exacerbation costs were from a more recent study conducted by Rubin et al. [26] were also used for this high-cost scenario.

#### Long-term efficacy of lumacaftor/ivacaftor

Assuming 100% efficacy of lumacaftor/ivacaftor in the first year, the efficacy for the remaining duration of the analysis was changed to 75%, 50% and 25% to reflect uncertainties in the long-term efficacy of the intervention.

#### Starting age of the cohort

Models were run by changing the starting age of the cohort to 6 years and 25 years. Age specific transition probabilities were derived from literature estimates of mean FEV1 levels using a bootstrapping method similar to the base case [5, 14]. Distributions of patients between mild, moderate and severe states (6 year old: 95%, 4%, 1%; 25 year old: 46%, 37%, 17%) were derived from the US CF Foundation Annual report, 2016 [17]. Other age-specific parameters including CF-specific mortality and probability of lung transplant and associated mortality were also estimated from literature [3, 17].

One-way sensitivity analysis was conducted for all model parameters including probabilities, utilities, costs, and treatment efficacy. The range of values for these variables were based on published literature. When data was unavailable, a range of ±20% of the point estimate was used for the analysis, including for the cost of lumacaftor/ivacaftor. A threshold analysis was also performed to determine the cost of the treatment required for it to be cost-effective at a threshold of $150,000 per QALY gained [27–29]. Finally, a probabilistic sensitivity analysis was conducted via a second-order Monte Carlo simulation of 10,000 samples. Beta distributions were used for the probabilities and utilities, gamma for costs and log-normal for relative risk.

## Results

### Lumacafor/Ivacaftor versus usual care (base-case analysis)

The results of the base-case analysis are shown in Table 3. Under the base-case, patients with CF who received usual care generated 6.84 QALYs and incurred $116,156 in costs during the 10-year horizon at a discount rate of 3%. Treatment with lumacaftor/ivacaftor resulted in 7.29 QALYs and costs of $1,778,921 over the 10 years, resulting in health gains of 0.45 QALYs and incremental cost of $1,662,765 compared with usual care. The resulting ICER of lumacafor/ivacaftor compared to usual care was $3,655,352 /QALY gained. For the usual care group, 48.2% of patients with CF remained in the “mild” Markov state, 30% in “moderate,” 5.9% in “severe,” 0.7% in “post-transplant,” and 15.1% died at the end of 10 years. This compares with 79.6% “mild”, 9.3% “moderate”, 0.1% “severe”, 0% “post-lung transplant” and 11% died at 10 years in the lumacafor/ivacaftor group.

**Table 3:** Results of base case and worst-case scenario analysis over a time horizon of two, four, six, eight and ten years

Scenario	Treatment	Cost ($)*	QALY	Incremental Cost ($)	Incremental Effectiveness (QALY gained)	ICER ($/QALY)
10-year time horizon						
Base-case	Usual care	116,156	6.84	1,662,765	0.45	3,655,352
	Lumacaftor/ivacaftor	1,778,921	7.29			
Worst-case	Usual care	116,156	6.84	1,677,901	0.20	8,480,265
	Lumacaftor/ivacaftor	1,794,056	7.04			
2-year time horizon						
Base-case	Usual care	30,469	2.23	531,606	0.07	7,311,801
	Lumacaftor/ivacaftor	562,075	2.3			
Worst-case	Usual care	30,469	2.23	532,262	0.06	9,292,285
	Lumacaftor/ivacaftor	562,731	2.28			
4-year time horizon						
Base-case	Usual care	51,850	3.56	852,463	0.15	5,835,535
	Lumacaftor/ivacaftor	904,313	3.70			
Worst-case	Usual care	51,850	3.56	855,802	0.09	9,554,343
	Lumacaftor/ivacaftor	907,652	3.65			
6-year time horizon						
Base-case	Usual care	72,361	4.77	1,148,863	0.24	4,869,328
	Lumacaftor/ivacaftor	1,221,224	5.00			
Worst-case	Usual care	72,361	4.77	1,155,718	0.12	9,263,760
	Lumacaftor/ivacaftor	1,228,079	4.89			
8-year time horizon						
Base-case	Usual care	94,274	5.86	1,418,488	0.34	4,173,169
	Lumacaftor/ivacaftor	1,512,761	6.20			
Worst-case	Usual care	94,274	5.86	1,429,656	0.16	8,861,944
	Lumacaftor/ivacaftor	1,523,930	6.02			

*QALY* Quality adjusted life year, *ICER* Incremental-cost effectiveness ratio

^*^All costs are expressed in USD-2016

### Worst-case scenario analysis

In worst-case scenario analysis, receiving lumacaftor/ivacaftor was associated with a gain of 7.04 QALYs and the costs increased to $1,794,056 for the 10-year time horizon (Table 3). The estimated differences in total 10-year QALYs, and costs for lumacaftor/ivacaftor compared to usual care were 0.20 and $1,667,900 respectively, resulting in ICER of $8,480,265/QALY gained. Compared to usual care (same distribution of patients as in base-case), 54% of people in the lumacaftor/ivacaftor group remained in the “mild” Markov state, 28.2% in “moderate,” 4.8% in “severe,” 0.5% in “post-transplant” and 12.6% in “death”.

### Other scenarios

**Fig. 2:**
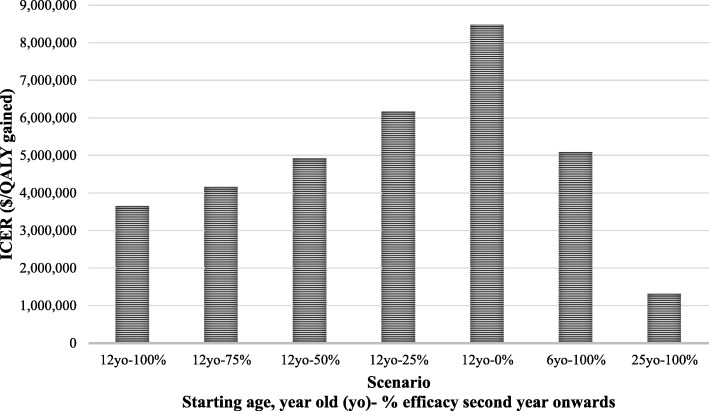
Scenario analysis results by changing vaccine efficacy and cohort starting age

The results of changing the base-case time period to two, four, six and eight years are shown in Table 3. The ICERs were higher in all of these shorter scenarios. For the high-cost scenario (i.e. increased costs for mild, moderate and severe health states,and higher pulmonary exacerbation costs), the base-case ICER dropped to $3,480,135/ QALY gained. The results of other scenarios altering long-term effectiveness and cohort starting age are shown in Fig. 2. In the 75%, 50% and 25% efficacy scenarios, the ICER increased to $4,168,163, $4,930,230 and $6,165,464/ QALY gained, respectively. Changing the starting age to 6 years led to an increase in ICER ($5,088,950/QALY gained), while changing it to 25 years resulted in a decrease ($1,321,306/QALY gained).

### Sensitivity analysis

**Fig. 3:**
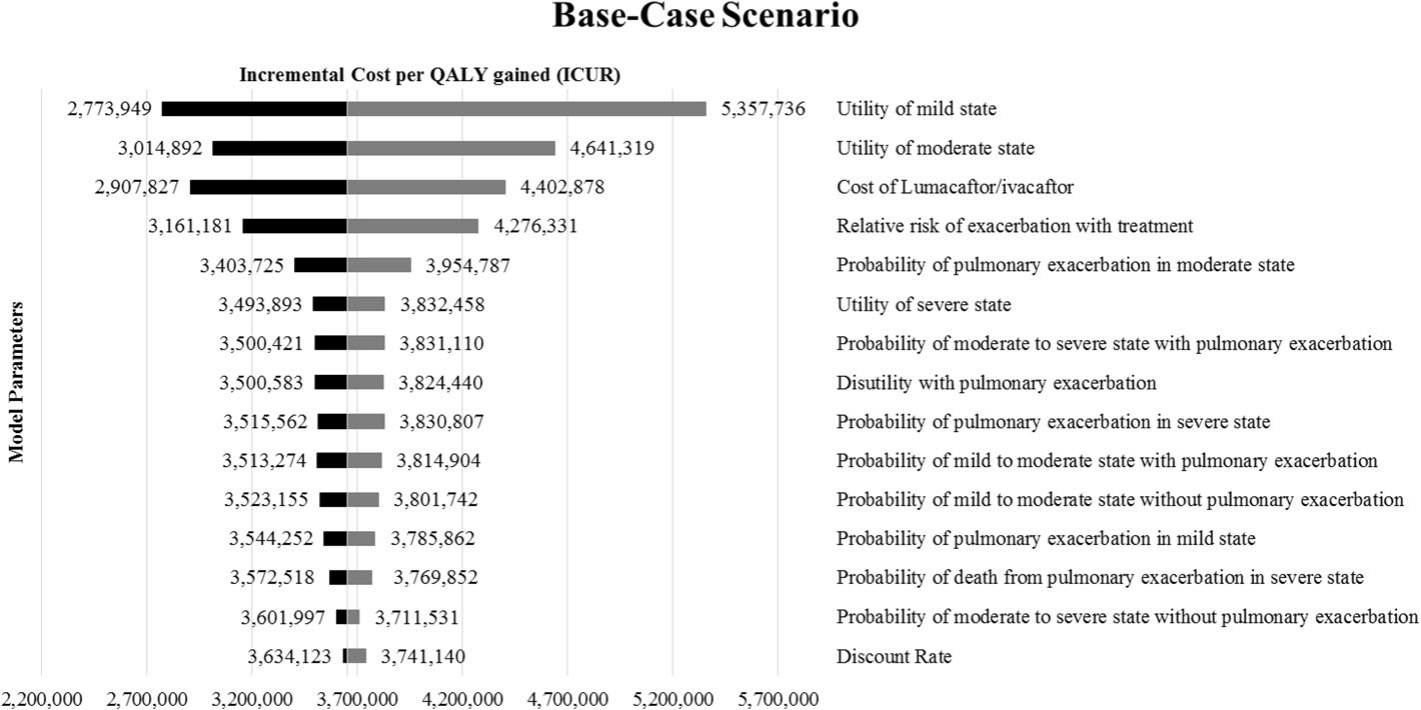
Results of one-way sensitivity analysis for base-case analysis

**Fig. 4:**
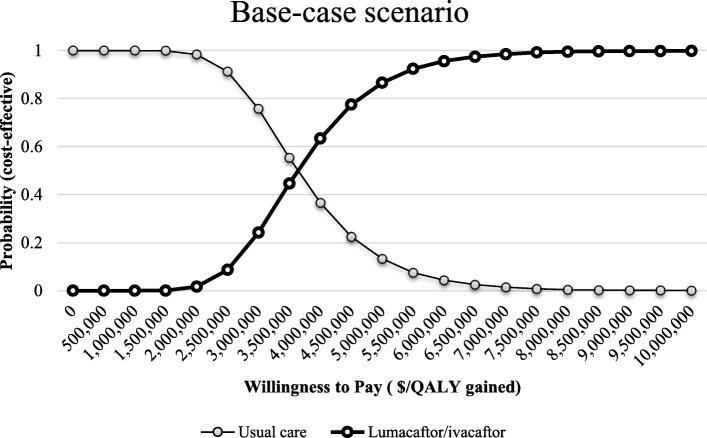
Cost-effectiveness acceptability curve for lumacaftor/ivacaftor vs usual care

Figure 3 shows the top 15 model inputs that substantially affected the base case results under one-way sensitivity analysis. Results of the threshold analysis suggest that lumacaftor/ivacaftor was cost-effective at a WTP threshold of $150,000/QALY when annual drug costs were lower than $4153. The cost-effectiveness acceptability curves in Fig. 4 show the percentage of iterations that were cost-effective at different WTP thresholds. For the base-case scenario, the probability of ivacaftor/lumacaftor combination being cost-effective compared to usual care was 0.8 or higher at a WTP of approximately $4,500,000/QALY gained.

## Discussion

The two phase 3 trials reported that lumacaftor/ivacaftor combination improved FEV1 levels and reduced the rate of pulmonary exacerbations in patients with CF who were homozygous for the F508del CFTR mutation. The studies concluded that this combination aimed to rectify the underlying cause of the disease by targeting CFTR and benefits patients homozygous for this gene mutation, who account for the 45% of patients with CF [5]. However, the high cost of this treatment and lack of long-term effectiveness data raises concerns about the drug’s long-term cost-effectiveness. The purpose of this study was to assess the cost-effectiveness of lumacaftor/ivacaftor combination for the treatment of 12 year-old patients with CF from the US payers’ perspective at a threshold of $150,000 per QALY gained for a time period of 10 years under differing assumptions of long-term effectiveness. The results of our study showed better health outcomes with the treatment, however, each additional QALY gained with the treatment compared to usual care was associated with an extremely high cost of over $3 Million. Among the different starting ages, the intervention was found to be most cost-effective in 25 year old population followed by 12 and 6. However, the ICER was still over a million dollars per QALY gained in all scenarios evaluated. This is well beyond typical cost-effectiveness thresholds considered acceptable in the US.

Two recently published observational studies assessed the outcomes associated with lumacaftor/ivacaftor in CF patients in routine clinical practice. The study by Labaste et al. examined acute changes in FEV1 levels post administration of the first dose of the treatment in 32 pediatric patients. The results of this study showed that there was a consistent decrease in patient FEV1 levels (− 10.4 ± 4.6%) [30]. A retrospective cohort study by Jennings et al. conducted at the John Hopkins CF center followed 116 patients with CF 1 year pre- and 11 months post the initiation of lumacaftor/ivacaftor therapy. The mean improvement in FEV1 was 0.11%, which is much lower than the 2.6–4.0% seen in the clinical trial. In addition, side effects were present in 39.7% of patients and a high rate of intolerance occurred with treatment. Both of these studies have a small sample size and show lowered effectiveness of the treatment than our worst-case scenario. This implies the incremental cost for each additional QALY gain could even be higher than estimated in our analysis [31].

A modeling study by Dilokthornsakul et al. estimated the lifetime clinical outcomes and cost of lumacaftor/ivacaftor compared to usual care in CF patients with a starting age of 25 years from a US health insurer’s perspective [10]. This compares with our study, which analyzed a variety of scenarios, including initiating lumacaftor/ivacaftor at ages 6, 12 and 25. The impact of lumacaftor/ivacaftor on patient outcomes was generally similar between our study and Dilokthornsakul et al., showing high treatment benefits and costs. For a starting age of 25 years, lumacaftor/ivacaftor was associated with a lifetime incremental cost of $2,632,249 and gain of 2.42 QALYs compared to our 10-year incremental values of $1,371,560 and 1.04 QALYs. These differences could be attributed to several factors. Their analysis was run over a lifetime compared to 10 years in our study, allowing patients to accrue more costs and QALY gains. Also, their analysis included a relatively healthier population for the starting age of 25 years. Their distribution of patients in mild, moderate and severe health states (72%, 25%, 3% respectively) was based on overall distribution for CF patients in the CF Foundation report and not just those aged 25, compared to the age-specific distribution in our analysis (46%, 37%, 17%). As a result of the biased inputs, the starting cohort in Dilokthornsakul et al. were more likely to live longer and accrue more costs as well benefits than a cohort aged 25 years at the model start. Despite these differences, the ICER estimated from their results, $1,087,706.2/QALY gained, is very close to the ICER estimated in our study for the same starting age ($1,321,306.35/QALY gained).

The ODA provided incentives for manufacturers to develop and market orphan drugs, including market exclusivity for 7 years, development tax credits, and a shorter approval period by FDA [32]. The total number of approved orphan drugs have increased from 34 before 1983 to 275 in 2009 [33]. Despite the availability of more treatment options for rare diseases, affordability is a major issue as many therapies with orphan status cost payers millions of dollars. Traditionally, there were no issues in coverage of these drugs because of smaller proportions of population associated with the use of these drugs. Payers have had very limited control when it comes to orphan drugs as many of these agents are the only available treatment options, rendering their demand insensitive to prices. However, the high rate of new orphan drug launches coupled with their increasing prices have resulted in payers increasing patient cost sharing as a method of cost-containment [7].

Our study has various strengths. Although the study by Dilokthornsakul et al. analyzed the lifetime costs and health outcomes associated with the treatment, this is the first study to estimate the cost-effectiveness of lumacaftor/ivacaftor under various scenarios and report the ICER associated with the treatment compared to usual care from the US payers’ perspective. Furthermore, a wide range of starting age for the intervention were employed in the analyses. The intervention was found to be more cost-effective when started in 25 year old patients than younger patients. While cost-effectiveness analyses are not explicitly used in the US for coverage decisions, insights from our study might help in understanding the value of this drug and its future use in different age-groups. In addition, the efficacy of the drug was modelled various scenarios. As more data becomes available on the effectiveness of this drug, the model can be updated and the cost-effectiveness reassessed.

There are a number of key assumptions and potential limitations to this study. First, with respect to the model structure, we chose a necessary, but simplified model to represent the severity of the disease as three major health states of mild, moderate and severe, rather than a continuum of severity. However, the model captures the efficacy of drug by allowing both FEV1 improvements and a reduction in the rate of pulmonary exacerbations.

Second, the patient population included in the model at cycle zero was defined as those 12 year-old with CF who were homozygous for the F508del CFTR mutation as the drug was first approved for this age group during the time of analysis. However, recently the drug was approved in patients 6 years and older. Thus, we conducted an additional analysis in a 6 year old cohort resulting in a higher ICER than the base case.

Third, input parameters such as the probability of moving from “mild” to “moderate” and “moderate” to “severe” health states and mortality associated with “mild” and “moderate” states were either indirectly derived from the literature when direct estimates were not available, or were based on expert opinion. However, univariate and probabilistic sensitivity analyses were performed on these parameters using broad ranges, and results show that these parameters had minimal impact on the resulting cost-effectiveness. Also, most model inputs were not specific to patients with F508del mutation as this data was not available at the time of developing this model. We anticipate that those with the F508del mutation would have worse outcomes than the modeled population, possibly resulting in larger absolute treatment differences and a better ICER than was estimated in our model.

Fourth, due to a lack of studies estimating the state specific costs for mild, moderate and severe disease, the cost estimates used for these health states and pulmonary exacerbations in the base case are based on a 1999 study. Given the significant advancement of CF care over the last two decades, these costs today might be much higher. To account for this, we re-estimated the results by using higher bounds of these costs in the model in the high-cost scenario. The resulting ICER was very similar to the base case.

Fifth, during the time of completion of this analysis, another CFTR modulator was approved in Feb 2017, tezacaftor/ivacaftor [34]. This drug combination is approved in the same patient population as lumacaftor/ivacaftor (two copies of F508) as well in patients with a single copy of 26 other mutations. The efficacy of this new drug combination is very similar to that of lumacaftor/ivacaftor in terms of its effect on mean absolute improvement in FEV1 levels (4% vs 4.3 to 6.7%), rate of pulmonary exacerbations (35% lower vs 30 to 39% lower than placebo) and rate of discontinuation due to adverse events (2.9% vs 4.2%) [5, 35]. As the comparator in trials of both these drugs was placebo with no head to head comparison and their prices are very similar, we believe its cost-effectiveness of this drug would be very similar to intervention in this current study. Also, to allow for patients switching to this newer drug, 2-year, 4-year, 6-year and 8-year time horizons were used to analyze the impact on results.

## Conclusion

In the future, denial of coverage to orphan drugs in the US seems unlikely [7]. However, as more of these agents are approved and enter the market with high prices, payers are more likely to closely analyze the situation and continue to attempt to discourage the use of high cost medications, while patient groups will be likely to advocate for access for all patients, and manufacturers continue to attempt to maximize profits. The introduction of new competitor therapies, such as tezacaftor/ivacaftor, may help to increase competition and make lumacaftor/ivacaftor more affordable [30]. A clear and deep understanding of the clinical and economic impact of these drugs by all the stake holders will play a pivotal role in decision making and pricing of these drugs. Given that lumacaftor/ivacaftor is used to a treat an orphan disease affecting a pediatric population, and is the first drug approved for patients with the F508del CFTR homozygous mutation, it gets privileges including marketing exclusivity and limited negotiating power by insurers. In the future, manufacturers, payers and other stake holders should work together for better pricing strategies, thereby ensuring cost-effective use of these agents.

## Abbreviations

CF: Cystic fibrosis
FDA: Food and drug administration
FEV: Forced expiratory volume
ICER: Incremental cost-effectiveness ratio
QALY: Quality adjusted life year
US: United States
